# Identification, characterization and preliminary X-ray diffraction analysis of the rolling-circle replication initiator protein from plasmid pSTK1

**DOI:** 10.1107/S1744309113023828

**Published:** 2013-09-28

**Authors:** Stephen B. Carr, Lauren B. Mecia, Simon E. V. Phillips, Christopher D. Thomas

**Affiliations:** a Research Complex at Harwell, Rutherford Appleton Laboratory, Didcot, Oxfordshire OX11 0FA, England; b Astbury Centre for Structural Molecular Biology, University of Leeds, Leeds LS2 9JT, England

**Keywords:** rolling-circle replication, plasmid pSTK1, plasmid pT181, replication initiation proteins, *Staphylococcus aureus*, *Geobacillus stearothermophilus*

## Abstract

Antibiotic resistance in bacterial pathogens poses an ever-increasing risk to human health. In antibiotic-resistant strains of *Staphylococcus aureus* this resistance often resides in extra-chromosomal plasmids, such as those of the pT181 family, which replicate *via* a rolling-circle mechanism mediated by a plasmid-encoded replication initiation protein. Currently, there is no structural information available for the pT181-family Rep proteins. Here, the crystallization of a catalytically active fragment of a homologous replication initiation protein from the thermophile *Geobacillus stearothermophilus* responsible for the replication of plasmid pSTK1 is reported. Crystals of the RepSTK1 fragment diffracted to a resolution of 2.5 Å and belonged to space group *P*2_1_2_1_2_1_.

## Abstract 2

A proteolytically stable fragment of a plasmid replication initiation protein from the thermophile *G. stearothermophilus* has been biochemically characterized, crystallized and diffraction data collected to a resolution of 2.5 Å.

## 1. Introduction

Bacterial plasmids provide a pool of antibiotic resistance determinants which may be exchanged among pathogens such as *Staphylococcus aureus* (Lyon & Skurray, 1987 ▶). Such plasmids can be broadly categorized into two groups: larger plasmids of 20 kb or greater, carrying multiple resistance determinants, or smaller plasmids of 5 kb or less which specify a single resistance determinant or may be cryptic (Novick, 1989 ▶). Among Gram-positive organisms these smaller plasmids are often found to replicate *via* a rolling-circle mechanism (Novick, 1989 ▶; del Solar *et al.*, 1998 ▶), whereby a plasmid-specified Rep protein makes a single-stranded cleavage at the replication origin, forming a transient, covalent protein–DNA adduct at the 5′ side of the nick permitting DNA synthesis by extension from the 3′ end. Rep proteins are also observed to nick and religate negatively supercoiled plasmid DNA containing the origin *in vitro* to form relaxed, covalently closed products (Koepsel *et al.*, 1985 ▶).

The sequences of plasmid Rep proteins have been compared with those involved in other rolling-circle processes, including phage and virus replication as well as conjugative DNA transfer (Koonin & Ilyina, 1993 ▶). These studies identified a major subgroup of such proteins, including the Rep_1 (PF01446) and Rep_2 (PF01719) families in the Pfam database (Punta *et al.*, 2012 ▶), which are characterized by a conserved HUH motif involved in binding the essential divalent metal ion. The structure of the RepB protein of pMV158, a representative of the Rep_2 family, has been solved (Boer *et al.*, 2009 ▶) and shows similarity to the relaxase domains of both viral (Hickman *et al.*, 2002 ▶) and conjugative transfer proteins (Boer *et al.*, 2006 ▶). Distinct from these examples, the Rep_trans (PF02486) family includes the Rep proteins encoded by plasmids of the staphylococcal pT181 family (Projan & Novick, 1988 ▶), as well as the conjugative functions of Tn916 and ICE*Bs*1 (Rocco & Churchward, 2006 ▶; Lee & Grossman, 2007 ▶). These proteins share less than 10% sequence identity with members of the Rep_2 family and lack the conserved HuH motif.

Studies of the initiator protein RepD (specified by the pT181-family member pC221) have identified the active-site tyrosine involved in forming the covalent linkage to DNA, and mutagenesis within this region has been used to identify further residues critical for activity (Thomas *et al.*, 1990 ▶, 1999 ▶). Crystals of proteins have been described previously for variants of the staphylococcal Rep proteins (Klimenko *et al.*, 1999 ▶); however, their structure solution was hampered by perfect merohedral twinning. In this study, we have turned our attention to the functional Rep protein of *Geobacillus stearothermophilus* plasmid pSTK1 (Narumi *et al.*, 1993 ▶; Nakayama *et al.*, 1993 ▶), a homologue of the staphylococcal proteins, with a view to understanding the function of the Rep_trans family proteins in molecular detail.

## 2. Materials and methods

### 2.1. Construction of Rep expression vectors

PCR amplification from pSTE33 (Narumi *et al.*, 1993 ▶; obtained from the RIKEN BRC, Japan) using primers BST343+ (5′-gggaattccatatgagtggtctgaagccttgtgt-3′) and PSTK-2 (5′-gccggatccgaattctattttgactgccgtataatc-3′) created a 343 amino-acid reading frame containing nucleotides 794–1822 of GenBank accession No. D29979. This product was cloned into pET15m (a variant of pET15b lacking *Eco*RI, *Cla*I and *Hin*dIII sites) *via* *Nde*I and *Bam*HI sites to give vector pET15m-Rep343, which was used for expression of RepSTK1 with a hexahistidine tag at the amino-terminus.

PCR amplification from pET15m-Rep343 using primers 15Nde (5′-ctggtgccgcgcggcagccatatg-3′) and QMPK (5′-GCCGGATCCGCAAGCTTACTActttggcatttgctttgcaatccg-3′) created a 269 amino-acid reading frame (residues 794–1597 of D29979) which was similarly cloned into pET15m to yield vector pET15m-RepQMPK and used for expression of residues 1–269 of RepSTK1 with a hexahistidine tag at the amino-terminus.

### 2.2. Expression and purification

*Escherichia coli* strain B834(λDE3)/pLysS cells were transformed with plasmid pET15m-Rep343 or pET15m-RepQMPK and grown to mid-log phase in LB medium containing 50 µg ml^−1^ ampicillin and 34 µg ml^−1^ chloramphenicol at 310 K. Protein expression was induced by the addition of isopropyl β-d-1-thiogalactopyranoside (IPTG) to a final concentration of 0.5 m*M* followed by incubation at 310 K for a further 3 h. Overexpressing cells were harvested by centrifugation for 10 min at 6000*g* and resuspended in 50 m*M* KH_2_PO_4_/K_2_HPO_4_ buffer pH 7.5, 500 m*M* KCl, 50 m*M* imidazole using 50 ml buffer for each litre of cell culture prior to sonication. The resuspension buffer also contained an EDTA-free protease-inhibitor cocktail tablet (Roche). Cells were lysed by sonication using an MSE Soniprep sonicator operating at an amplitude of 8 µm. The sonicated cell suspension was warmed to 338 K for 15 min followed by the removal of cell debris by centrifugation at 22 000*g* for 30 min at 288 K. The supernatant was applied onto a 5 ml HisTrap Column (GE Healthcare, Amersham, England) pre-equilibrated with resuspension buffer followed by extensive washing with the same buffer. RepSTK1 or RepSTK1_(1–269)_ was eluted from the column using a 50–500 m*M* gradient of imidazole over 20 column volumes with protein elution monitored by absorbance at 280 nm. Protein-containing fractions were pooled and dialysed against 50 m*M* Tris–HCl pH 7.5, 200 m*M* KCl, 10%(*v*/*v*) ethanediol.

Protein was precipitated by the addition of 2.3 *M* ammonium sulfate and incubation at 277 K for 30 min with gentle mixing, followed by centrifugation at 12 000*g* for 30 min and resuspension in 15 ml 50 m*M* Tris–HCl pH 7.5, 1 m*M* EDTA, 10%(*v*/*v*) ethanediol (buffer K0). Further buffer K0 was added until the final conductivity of the buffer containing the resuspended sample matched that of 50 m*M* Tris–HCl pH 7.5, 200 m*M* KCl, 1 m*M* EDTA, 10%(*v*/*v*) ethanediol (buffer K200). A 5 ml Q Sepharose column (GE Healthcare, Amersham, England) was connected in series to a 5 ml Heparin HP column (GE Healthcare, Amersham, England) and equilibrated with buffer K200. The resuspended RepSTK1 sample was then applied onto these columns, Q Sepharose first, followed by washing with at least ten column volumes of K200. After washing, the Q Sepharose column was removed from the circuit and RepSTK1 was eluted by the application of a 200 m*M*–1 *M* gradient of KCl over 20 column volumes in the same buffer. Protein elution was monitored by absorbance at 280 nm and protein-containing fractions were analysed by SDS–PAGE.

For samples subject to crystallization, the hexahistidine tag was removed from RepSTK1_(1–269)_ by thrombin cleavage prior to the ammonium sulfate precipitation and Heparin Sepharose chromatography steps. Thrombin was added to a final concentration of 30 units per 10 ml of partially purified protein, followed by incubation at room temperature for 2 h. Cleavage was assessed by SDS–PAGE to ensure complete removal of the tag. Purification then proceeded as described above.

Analytical gel filtration of purified RepSTK1_(1–269)_ was performed by loading 50 µl protein solution onto a Superdex S75 10/300 column (GE Healthcare, Amersham, England) pre-equilibrated with 50 m*M* Tris–HCl pH 7.5, 500 m*M* KCl. Multi-angle laser light-scattering (MALLS) data were collected by passing the column eluate through a DAWN HELIOS II light-scattering system (Wyatt Technology Corporation, Santa Barbara, USA) connected in series to the column and analysed using the *ASTRA* software package.

### 2.3. Partial proteolysis and characterization of products

RepSTK1 (10 µg) was digested with either 0.01, 0.1 or 1 µg pronase from *Streptomyces griseus* (Sigma, UK) in 20 µl buffer K200 at 310 K for 30 min. Products were separated by SDS–PAGE before submission to the peptide mass-fingerprinting service at the University of Leeds.

### 2.4. Activity assays

The double-stranded origin of pSTK1 (within nucleotides 640–874 of D29979) was amplified from pSTE33 by PCR using primers X+ (5′-GGGTCTAGACCGGCACCAGCCGAC-3′) and P− (5′-GGGCTGCAGTTTTTCAACGCATTTTTTTACCG-3′) and cloned into vector pCER19 (Caryl *et al.*, 2004 ▶) *via* the *Xba*I and *Pst*I sites to create plasmid pCER*oriSTK1*. Negatively supercoiled pCER*oriSTK1* was purified by density-gradient centrifugation in caesium chloride/ethidium bromide.

Topoisomerase assays contained either RepSTK1 or RepSTK1_(1–269)_ at concentrations of 4–64 n*M* (calculated as a dimer) combined with 0.5 µg of negatively supercoiled pCER*oriSTK1* in 30 µl K200 buffer containing 10 m*M* MgCl_2_. After incubation at 338 K for 1 h reactions were terminated by the addition of 4 µl dye/EDTA and products were separated by electrophoreses in the presence of ethidium bromide as described previously (Thomas *et al.*, 1995 ▶).

### 2.5. Crystallization and data collection

Prior to crystallization, RepSTK1_(1–269)_ was dialysed against 50 m*M* Tris–HCl pH 7.5, 700 m*M* KCl and concentrated to 5 mg ml^−1^ using Amicon Ultra centrifugal concentrators (Merck Millipore, Watford, England) containing a 10 kDa molecular-weight cutoff membrane. The protein concentration was estimated from the sample absorbance at 280 nm using an extinction coefficient of 1.369 *M* ^−1^ cm^−1^. After concentration the protein could be stored at 277 K prior to crystallization if necessary. Crystals of RepSTK1_(1–269)_ were grown using the sitting-drop vapour-diffusion method by mixing 100 nl protein solution with 100 nl crystallization buffer using a Cartesian MicroSys crystallization robot (Digilab Ltd, Huntingdon, England). The initial crystallization screens tested were Index (Hampton Research, USA) and Morpheus (Molecular Dimensions Ltd, UK) followed by incubation at 293 K. Crystals grew from buffer consisting of 0.1 *M* HEPES pH 7.5, 10%(*v*/*v*) PEG 3350, 0.2 *M* proline (Index condition No. 61) within 24 h and no further optimization of crystallization was performed.

Prior to data collection, crystals were cryoprotected by the addition of 25%(*v*/*v*) glycerol to a stabilizing solution consisting of 0.1 *M* HEPES pH 7.5, 12%(*v*/*v*) PEG 3350, 0.2 *M* proline followed by flash-cooling in liquid nitrogen. Diffraction data were collected at a temperature of 100 K at station I04-1 of the Diamond Light Source (DLS), UK at a wavelength of 0.9163 Å using a PILATUS 2M hybrid pixel-array detector. The crystal-to-detector distance was 325 mm and 1800 diffraction images were collected, each with an oscillation range of 0.2°. Data reduction was performed using *iMOSFLM* (Battye *et al.*, 2011 ▶) and *AIMLESS* (Evans, 2011 ▶), with any additional analysis being performed using programs from the *CCP*4 suite (Winn *et al.*, 2011 ▶).

## 3. Results and discussion

**Figure 1:**
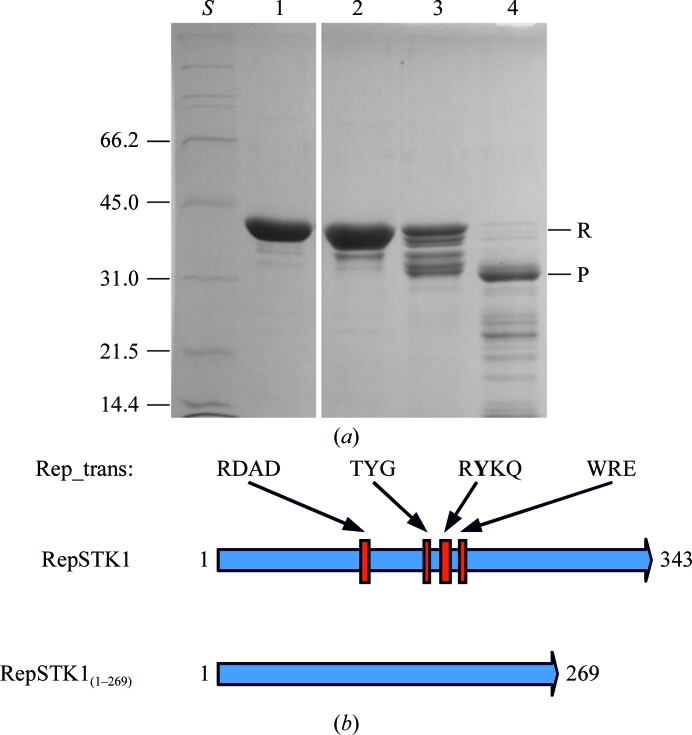
(*a*) SDS–PAGE of RepSTK1 following digestion of RepSTK1 with pronase. Lane *S*, size markers (pertinent masses are given on the left in kDa); lane 1, RepSTK1 (no digestion); lanes 2, 3 and 4, RepSTK1 digested with 0.01, 0.1 and 1 µg pronase, respectively. R shows the position of the undigested protein, P shows the protease-resistant fragment. (*b*) Location of the Rep_trans motif with the RepSTK1 reading frame and extent of the truncated form RepSTK1_(1–269)_ crystallized in this study.

RepSTK1 was expressed in *E. coli* as a 343 amino-acid protein derived from the original pSTK1 sequence fused to a hexahistidine tag at the N-terminus. Limited proteolysis of RepSTK1 revealed a protease-resistant fragment of approximately 31 kDa (Fig. 1 ▶ *a*). Characterization of this fragment by peptide mass fingerprinting identified tryptic fragments corresponding to residues 1–262 of RepSTK1, which includes the conserved residues of the Rep_trans motif (Fig. 1 ▶ *b*).

**Figure 2:**
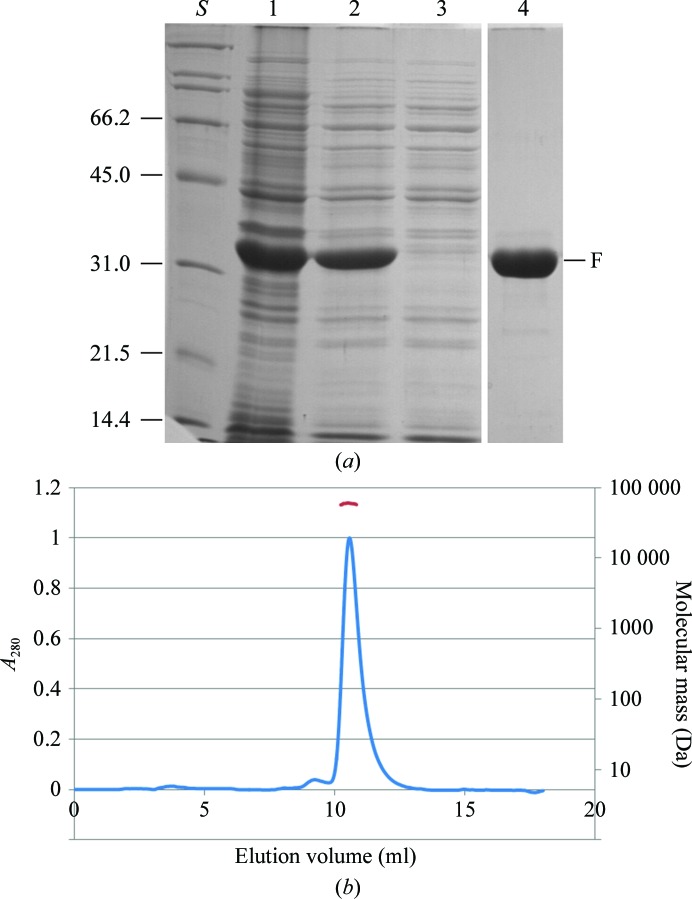
(*a*) SDS–PAGE following purification of RepSTK1_(1–269)_. Lane *S*, size markers (as Fig. 1 ▶); lane 1, whole cells following induction of expression with IPTG; lane 2, cleared lysate prior to loading the HisTrap column; lane 3, unbound material; lane 4, peak fraction following elution from HisTrap column. F shows the position of the purified fragment. Further purification steps were required to remove nonspecific nuclease activity from the protein preparations, but did not improve the purity of the sample as assessed by SDS–PAGE. (*b*) SEC–MALLS trace for RepSTK1_(1–269)_ monitored by absorbance at 280 nm (blue). The protein elutes at a volume of 10.5 ml corresponding to an apparent molecular mass of 60 kDa and the absolute molecular mass (red) calculated from the MALLS signal corresponds to a mass of 62 kDa.

**Figure 3:**
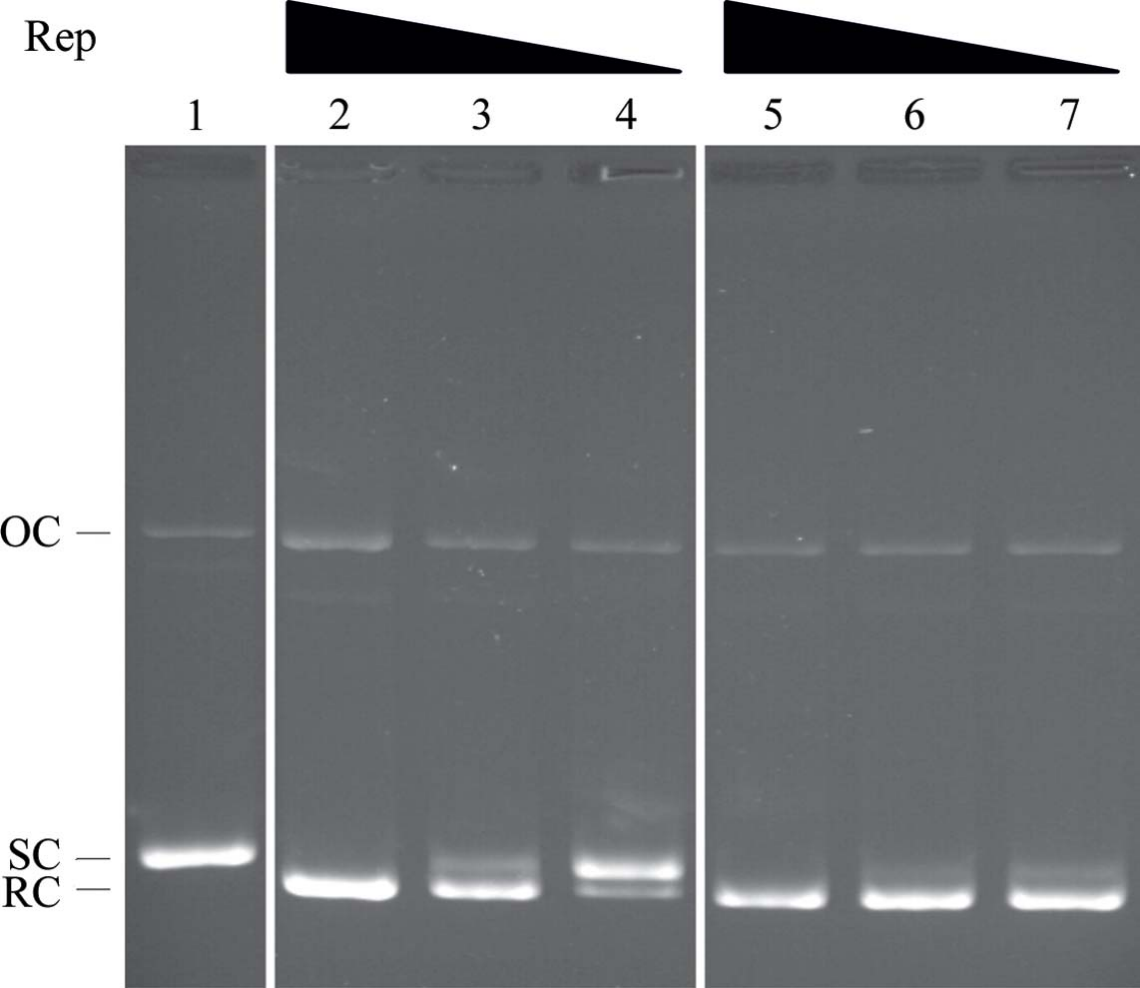
Agarose gel showing the topoisomerase activity of Rep proteins. Lane 1, negatively supercoiled pCER*oriSTK1*; lanes 2, 3 and 4, plasmid after incubation with 64, 16 and 4 n*M* RepSTK1, respectively; lanes 5, 6 and 7, plasmid after incubation with 64, 16 and 4 n*M* RepSTK1_(1–269)_, respectively. SC, negatively supercoiled substrate DNA; OC, nicked open-circular intermediate; RC, relaxed covalently closed product.

Variants of RepSTK1 were designed with stop codons at different locations in this region, of which RepSTK1_(1–269)_ (consisting of residues 1–269 of RepSTK1 fused to a hexahistidine tag) expressed at a high level and was readily purified (Fig. 2 ▶ *a*) with a yield of 50 mg protein per litre of culture. Size-exclusion chromatography (SEC) (Fig. 2 ▶ *b*) shows that the protein migrates as a single peak with an apparent molecular mass of 60 kDa, while the absolute molecular mass calculated from the MALLS data is 62 kDa, both suggesting that the protein exists as a dimer in solution. This reflects the native state of the staphylococcal Rep proteins, which are also dimeric (Thomas *et al.*, 1990 ▶). Like RepSTK1, the truncated RepSTK1_(1–269)_ displays sequence-specific topoisomerase-like activity typically observed in this family of proteins (Fig. 3 ▶), converting negatively supercoiled plasmid containing the pSTK1 origin of replication into a relaxed, covalently closed form.

**Figure 4:**
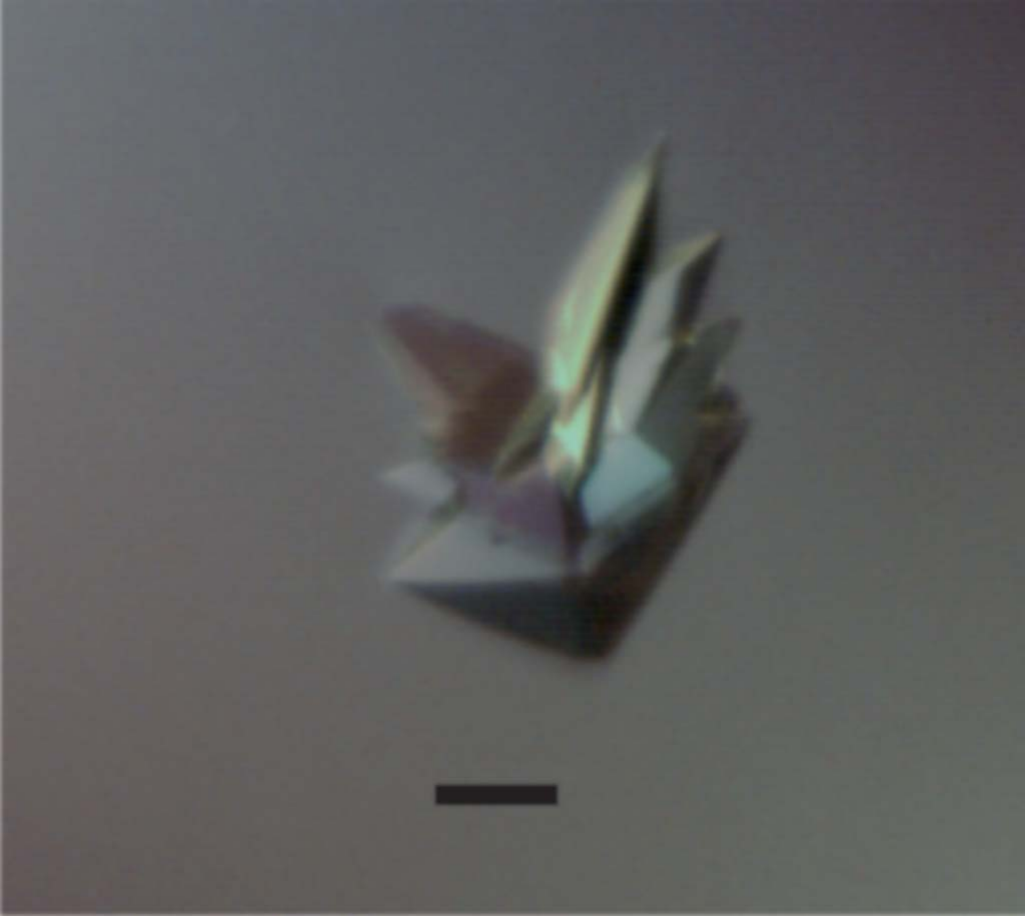
Image of typical RepSTK1_(1–269)_ crystals grown by sitting-drop vapour diffusion. The scale bar represents a length of 100 µm.

Removal of the hexahistidine tag was necessary for the growth of crystals that diffracted to high resolution. After thrombin cleavage, three amino acids (GSH) remained at the N-terminus of residues 1–269 of RepSTK1. Crystals grew as clusters of thick plates (Fig. 4 ▶); these could be easily separated with an acupuncture needle and each individual crystal grew to average dimensions of 250 × 150 × 50 µm. X-ray diffraction data were collected to a maximum resolution of 2.5 Å (Table 1 ▶) and subsequent reduction suggested that the crystals belonged to space group *P*2_1_2_1_2_1_, with unit-cell parameters *a* = 66.3, *b* = 137.1, *c* = 149.3 Å. Calculation of the Matthews coefficient (Matthews, 1968 ▶) using a sequence-based molecular weight of 31 391 Da resulted in a *V*_M_ of 2.69 Å^3^ Da^−1^ and a solvent content of 54.9%, assuming the presence of four molecules per asymmetric unit. Owing to the absence of any structural homologues, experimental phasing methods will be used to aid the structure determination of this protein.

## Figures and Tables

**Table 1:** X-ray data-collection statistics Values in parentheses are for the outermost resolution shell.

X-ray source	I04-1, DLS
Space group	*P*2_1_2_1_2_1_
Unit-cell parameters (Å)	*a* = 66.3, *b* = 137.1, *c* = 149.3
Wavelength (Å)	0.9163
Temperature (K)	100
Resolution (Å)	74.6–2.5 (2.57–2.50)
Total reflections	628790 (48471)
Unique reflections	47781 (3510)
Completeness (%)	99.9 (100)
Multiplicity	13.2 (13.8)
〈*I*/σ(*I*)〉	25.3 (3.3)
*R*_merge_ † (%)	5.7 (84)
*R*_meas_ ‡ (%)	6.1 (91)
*R*_p.i.m._ § (%)	1.7 (24.4)
Matthews coefficient (Å^3^ Da^−1^)	2.69
Solvent content (%)	54.9
Molecules per asymmetric unit	4

† *R*_merge_ = 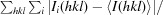 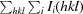, where *I_i_*(*hkl*) is the intensity of reflection *hkl* and 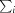 is the sum over all *i* measurements of reflection *hkl*.

‡ *R*_meas_ = 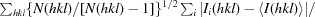 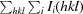, where *I_i_*(*hkl*) is the intensity of reflection *hkl*, 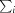 is the sum over all *i* measurements of reflection *hkl* and *N*(*hkl*) is the multiplicity of reflction *hkl.*

§ *R*_p.i.m._ = 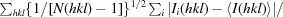 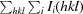, where *I_i_*(*hkl*) is the intensity of reflection *hkl*, 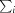 is the sum over all *i* measurements of reflection *hkl* and *N*(*hkl*) is the multiplicity of reflction *hkl.*
